# Supplementary material for: Isolation, In Vitro and In Silico Anti-Alzheimer and Anti-Inflammatory Studies on Phytosteroids from Aerial Parts of Fragaria × ananassa Duch
Source: Biomolecules. 2022 Oct 6;12(10):1430. doi: 10.3390/biom12101430 (PMC9599613; doi:10.3390/biom12101430)

# Supporting Information

Figure S1: $^{1}H$ NMR spectrum of compound 1.

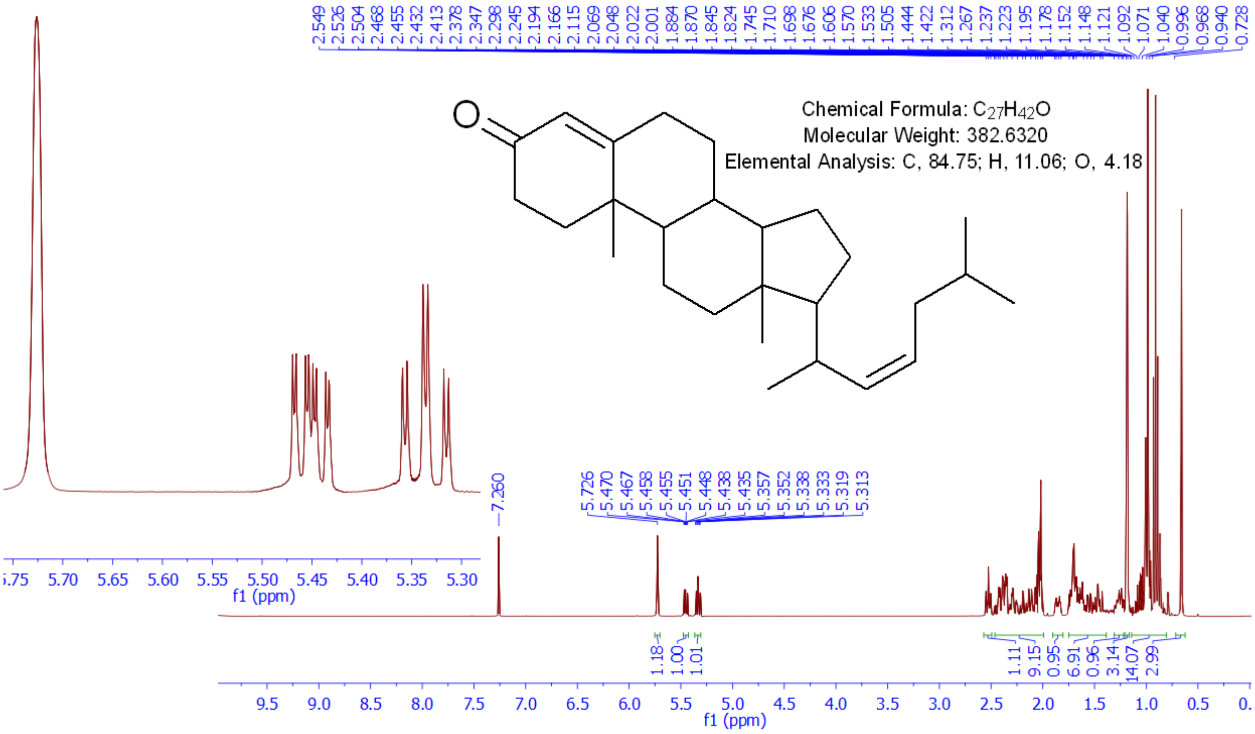

Table S1: GC-MS details of Compound 1.

| Compound Label | Name | *m/z* | RT | Algorithm |
|---|---|---|---|---|
| 4,22-Cholestadien-3-one $$ (22Z)-Cholesta-4,22-dien-3-one # | **4,22-Cholestadien-3-one $$ (22Z)-Cholesta-4,22-dien-3-one #** | 55.1 | 50.196 | Find by Chromatogram Deconvolution |

Figure S2: GC-MS chromatogram of Compound 1.

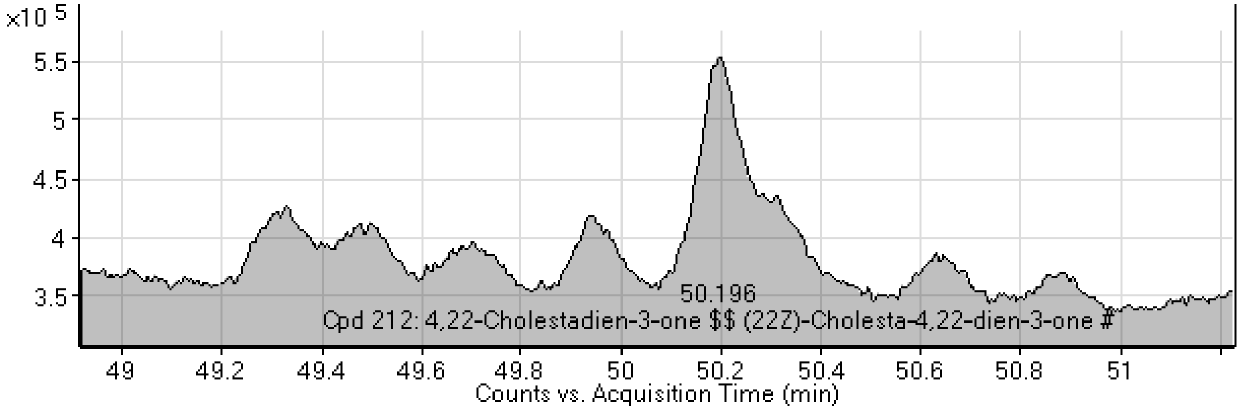


Figure S3: MS spectrum and integration pattern of compound 1.

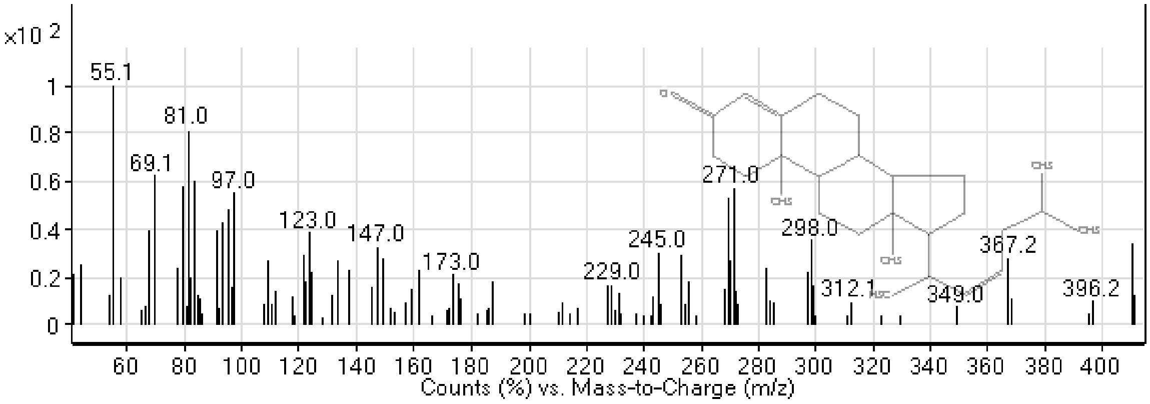

Figure S4: MS zoomed spectrum of compound 1.

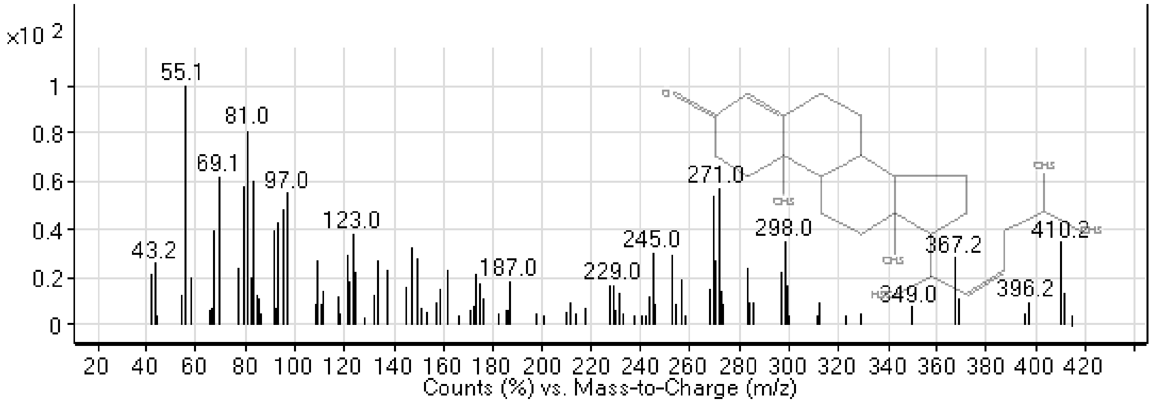


Table S2: MS spectrum peak list of compound 1.

| *m/z* | **Abund** |
|---|---|
| 55.1 | 13151.6 |
| 69.1 | 8198.9 |
| 79.1 | 7667.9 |
| 81 | 10749.9 |
| 83.1 | 7854.9 |
| 93 | 5692.3 |
| 95 | 6430.1 |
| 97 | 7281.4 |
| 269 | 7034.3 |
| 271 | 7601.2 |

Figure S5: Library spectrum of compound 1.

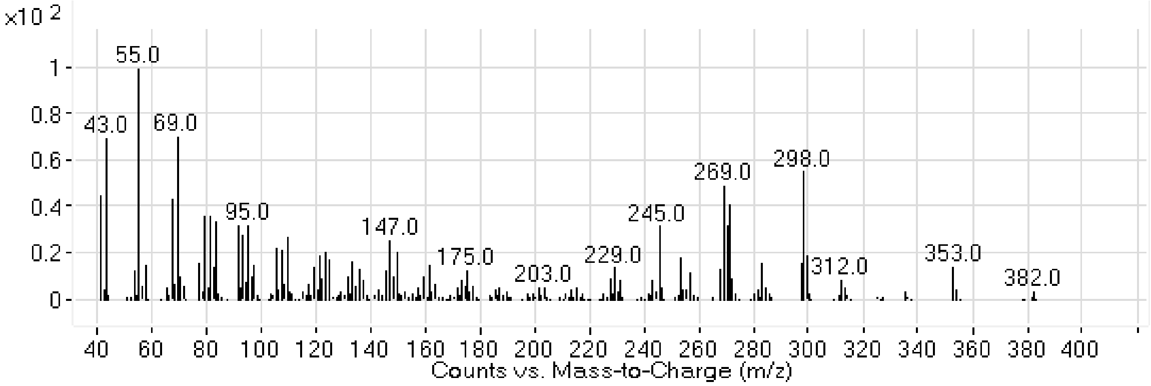

Figure S6: Difference spectrum of compound 1.

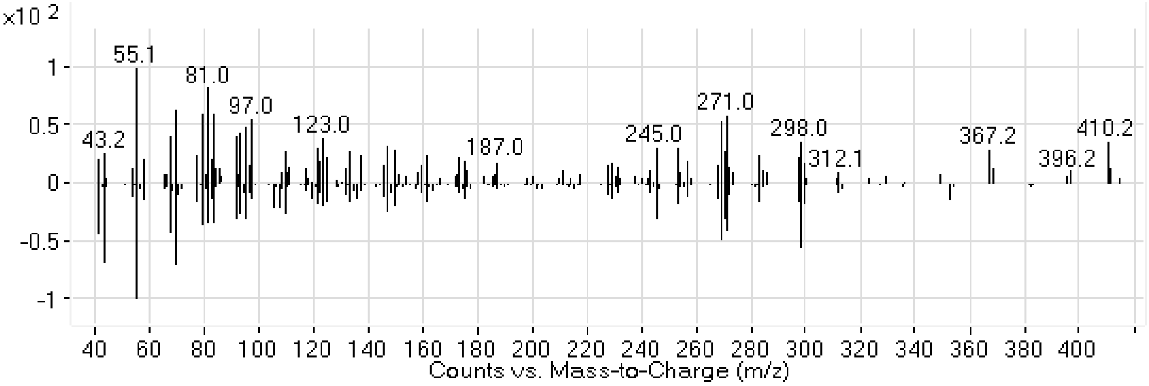


Figure S7: $^{1}$H NMR spectrum of compound 2.

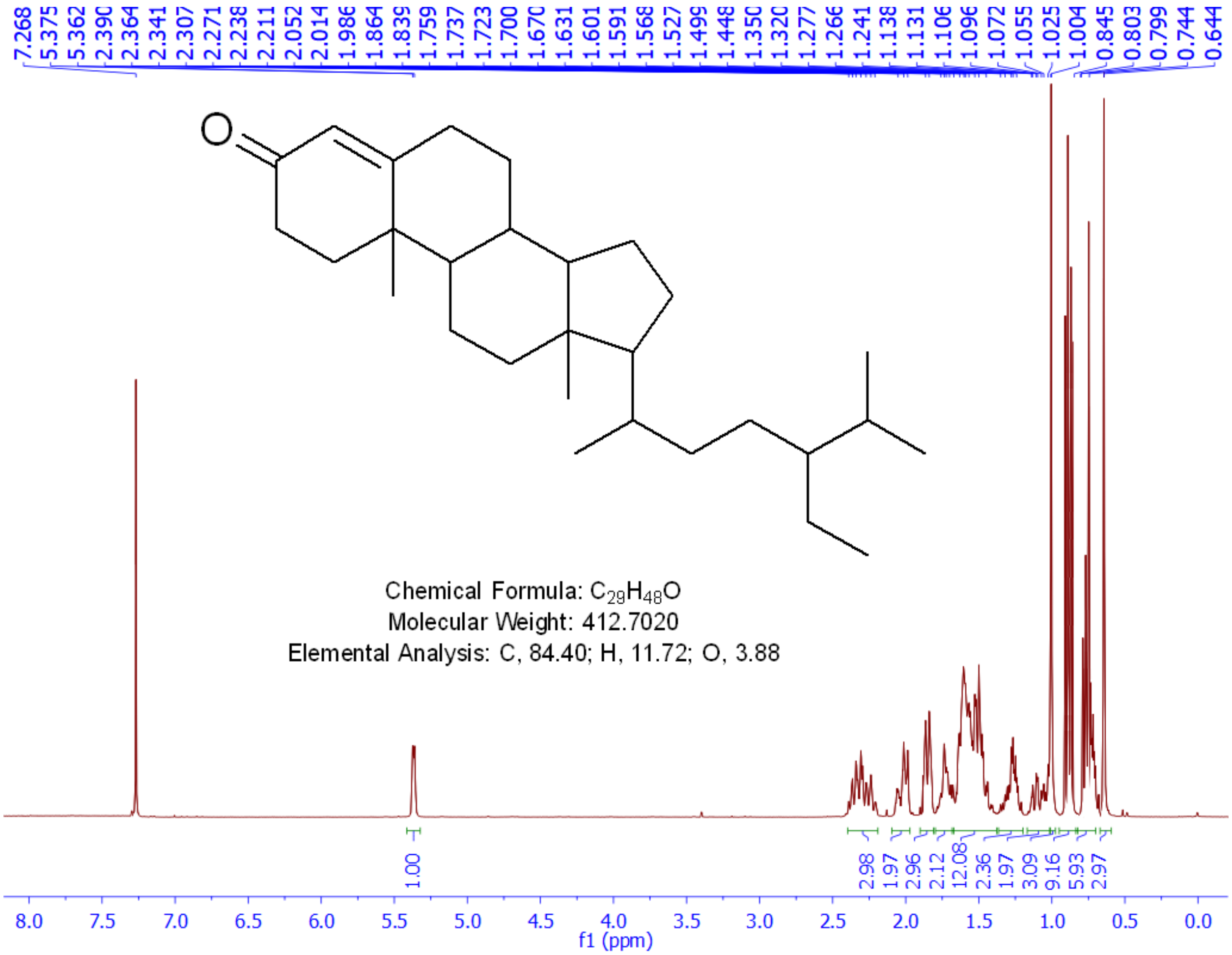

Table S3: GC-MS details of compound 2.

| Compound Label | Name | *m/z* | RT | Algorithm |
|---|---|---|---|---|
| Stigmast-4-en-3-one $$ 4-Stigmasten-3-one $$ Sitostenone | **Stigmast-4-en-3-one $$ 4-Stigmasten-3-one $$ Sitostenone** | 124 | 51.625 | Find by Chromatogram Deconvolution |

Figure S8: GC-MS chromatogram of compound 2.

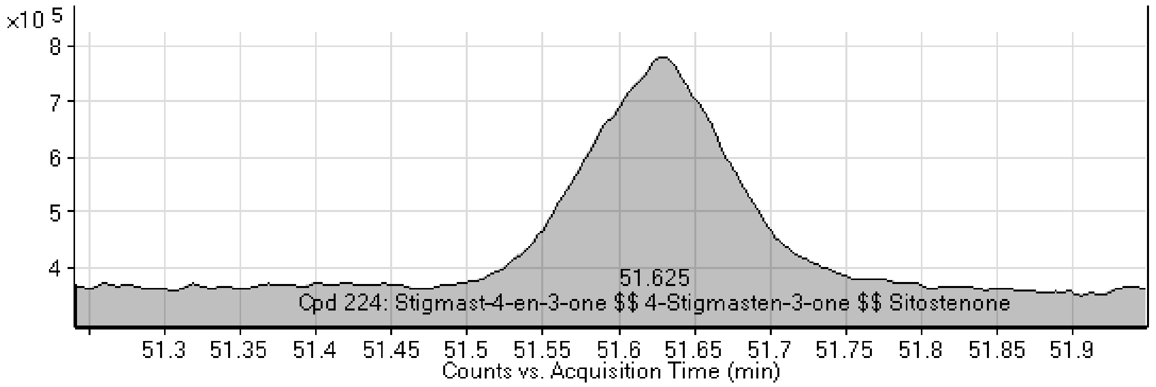


Figure S9: MS spectrum of compound 2.

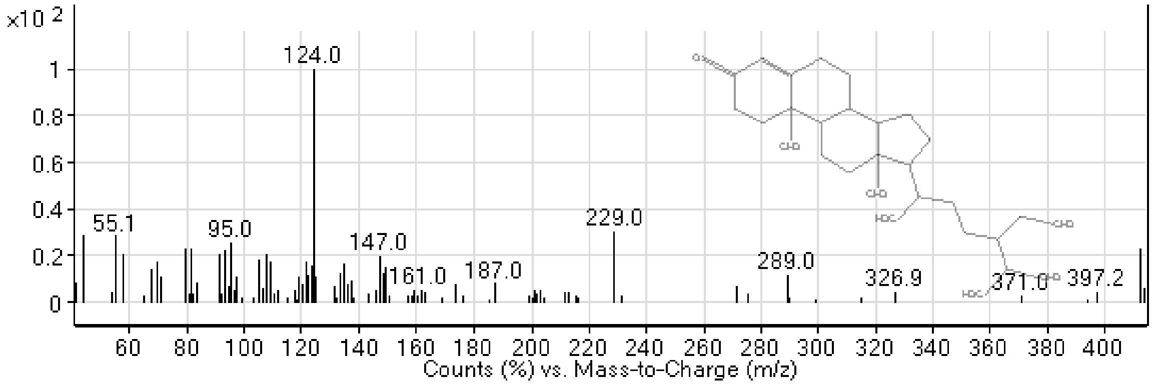

Figure S10: Zoomed spectrum of compound 2.

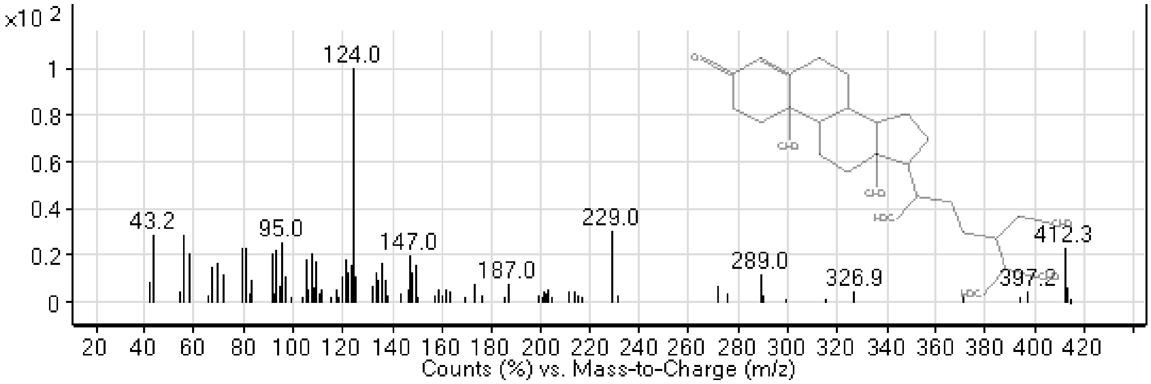


Table S4: MS spectrum peak list of compound 2.

| *m/z* | Abund |
|---|---|
| 43.2 | 14781 |
| 55.1 | 14639.6 |
| 57.1 | 10830.3 |
| 79.1 | 11955.5 |
| 81 | 11873.6 |
| 93 | 11429.3 |
| 95 | 13110 |
| 124 | 50679.9 |
| 229 | 15629.7 |
| 412.3 | 11847.4 |

Figure S11: Library spectrum of compound 2.

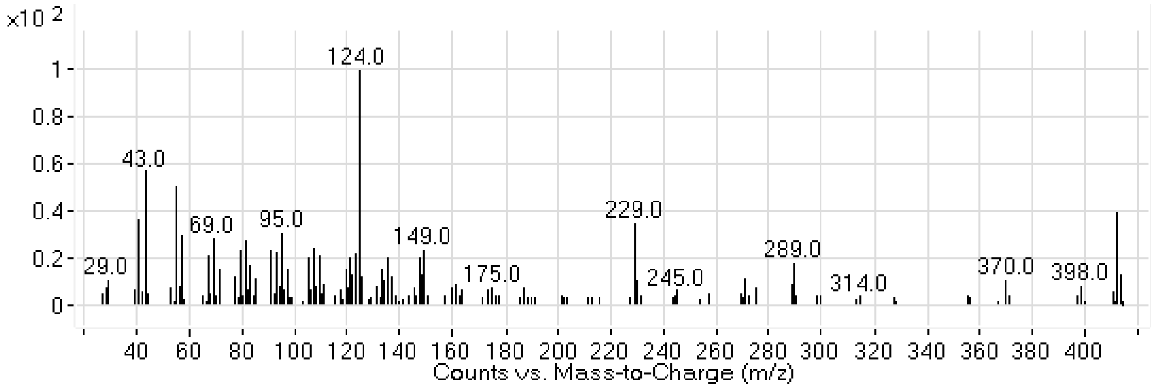

Figure S12: Difference spectrum of compound 2.

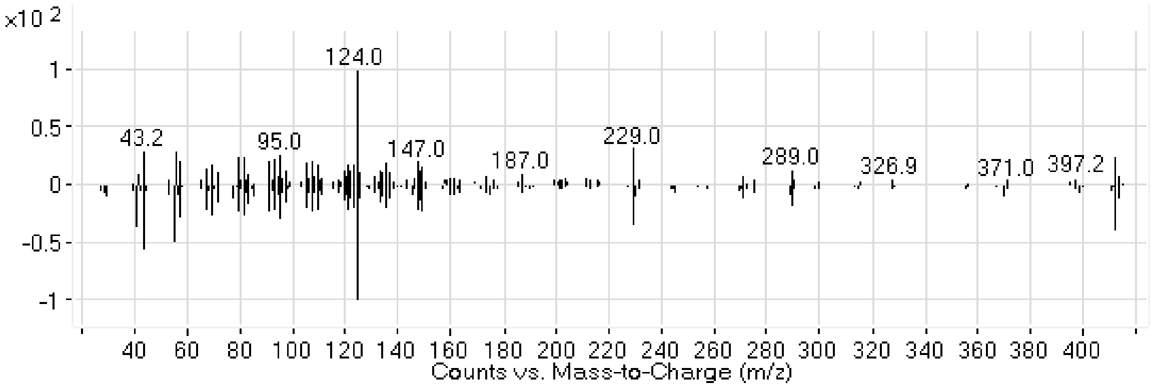

Supplement: Supplementary file 1 [file biomolecules-12-01430-s001.zip › biomolecules-1849934-supplementary.pdf]
